# Childhood ODD and ADHD Behavior: The Effect of Classroom Sharing, Gender, Teacher Gender and Their Interactions

**DOI:** 10.1007/s10519-015-9712-z

**Published:** 2015-02-25

**Authors:** Eveline L. de Zeeuw, Catharina E. M. van Beijsterveldt, Gitta H. Lubke, Tina J. Glasner, Dorret I. Boomsma

**Affiliations:** 1 Department of Biological Psychology, VU University, Van der Boechorststraat 1, 1081 BT Amsterdam, The Netherlands; 2 EMGO+ Institute for Health and Care Research, VU University Medical Centre, Amsterdam, The Netherlands; 3 Department of Psychology, University of Notre Dame, Notre Dame, IN USA

**Keywords:** ODD, ADHD, Conners’ Teacher Rating Scales, Measurement invariance, Heritability

## Abstract

One criterion for a diagnostic and statistical manual of mental disorders (DSM-IV) diagnosis of attention deficit hyperactivity disorder (ADHD) and oppositional defiant disorder (ODD) is that symptoms are present in at least two settings, and often teacher ratings are taken into account. The short Conners’ Teacher Rating Scales—Revised (CTRS-R) is a widely used standardized instrument measuring ODD and ADHD behavior in a school setting. In the current study CTRS-R data were available for 7, 9 and 12-year-old twins from the Netherlands Twin Register. Measurement invariance (MI) across student gender and teacher gender was established for three of the four scales (Oppositional Behavior, Hyperactivity and ADHD Index) of the CTRS-R. The fourth scale (ATT) showed an unacceptable model fit even without constraints on the data and revision of this scale is recommended. Gene-environment (GxE) interaction models revealed that heritability was larger for children sharing a classroom. There were some gender differences in the heritability of ODD and ADHD behavior and there was a moderating effect of teacher’s gender at some of the ages. Taken together, this indicates that there was evidence for GxE interaction for classroom sharing, gender of the student and gender of the teacher.

**Electronic supplementary material:**

The online version of this article (doi:10.1007/s10519-015-9712-z) contains supplementary material, which is available to authorized users.

## Introduction

Attention deficit hyperactivity disorder (ADHD) is characterized by difficulties of both inattention and hyperactivity or impulsiveness that interfere with a child’s daily functioning. At school, children have, for example, difficulty remaining in their seats and paying attention for a longer period of time. Oppositional defiant disorder (ODD) is characterized by hostile and defiant behavior towards figures with authority, going beyond normal childhood behavior. Children argue with their teacher and often lose their temper (American Psychiatric Association 2000). Numerous studies have found a negative association between ADHD and educational achievement (Polderman et al. 2010) and children with ODD receive lower grades at school (Greene et al. 2002). Both children with ADHD and ODD are more likely to attend specialized schools.

The American Psychiatric Association (APA) estimates that 3–7 % of all school-aged children are diagnosed with ADHD, while estimates of the prevalence of ODD in children range from 2 to 16 % (American Psychiatric Association 2000). It must be noted that more than 50 % of the children diagnosed with ADHD also have ODD (Angold et al. 1999; Wilens et al. 2002). In the general population, the ratio between boys and girls with ADHD is estimated to be 3:1, while the ratio is higher in a clinical population (Gaub and Carlson 1997). A potential explanation of the discrepancy in the ratio between boys and girls on population versus clinical level is bias in the ratings of the teacher (Abikoff et al. 2002; Derks et al. 2007b; Sciutto et al. 2004), because one criterion for a diagnostic and statistical manual of mental disorders (DSM-IV) diagnosis is that symptoms are present in at least two settings and often the evaluation of the teacher is taken into account. In a study focusing on children diagnosed with ADHD (Derks et al. 2007b) teachers reported more disruptive behavior at school for boys than for girls, while there is no difference for mother ratings. For ODD, teachers also report higher prevalence rates in boys than girls while parents do not (Meisel et al. 2013). To further complicate matters, teacher bias may depend on the teacher’s gender. An alternative explanation of the discrepancy is that the gender differences in ADHD and ODD behavior are more pronounced in the school environment, which may demand more of a child than the home environment.

When analyzing questionnaire data concerning psychiatric disorders, researchers often use sum scores to combine multiple items of a scale. A meaningful interpretation of a sum score is only possible when a scale measures the same disorder in all specified groups. Mellenberg (1989) defined measurement invariance (MI) with respect to group as an identical distribution of the observed sum score, conditional on the disorder that the test measures, across groups. The interpretation of group differences with respect to sum scores is only meaningful when the scale is MI (Slof-Op ‘t Landt et al. 2009). MI does not hold for example if boys score on average higher on some of the items than girls without actually scoring higher on the underlying disorder. In this case, a boy and girl, who have the same degree of a disorder, obtain systematically different sum scores. Group differences in the sum score will then reflect measurement bias instead of true underlying differences (Dolan 2000; Mellenbergh 1989; Meredith 1993; Millsap and Yun-Tein 2004).

Behavioral genetic studies have established that ADHD is amongst the most heritable psychiatric childhood disorders. According to a review of 20 twin studies, the mean estimate of the heritability of ADHD in children is over 75 % (Faraone et al. 2005). Estimates for ODD are somewhat lower with a heritability of around 50 % (Hudziak et al. 2005). Heritability estimates of problem behavior in primary school children vary widely between twins taught in the same classroom compared to twins with different teachers (Saudino et al. 2005). It is a general finding that twin correlations are larger when one teacher rates both children compared to when two teachers each rate one child. One hypothesis is that ratings could be biased due to the same person rating both children when twins are taught in the same classroom. Each teacher has his or her own perception on behavior, which can make children seem more similar when they have the same teacher (Kan et al. 2013; Simonoff et al. 1998). The second hypothesis is that there is gene-environment (GxE) interaction (Eaves 1984), which holds that the variation in the behavior of children in different classroom environments may depend on their genetic make-up. The classroom environment, teacher characteristics and peers differ when the twins do not share a classroom in primary school, and different environments might trigger different behavior depending on a child’s genes. A study of internalizing and externalizing behavior in primary school children concluded that this was the case, and that the heritability was higher in children sharing a classroom compared to children in different classrooms because of GxE interaction (Lamb et al. 2012). The question is whether this is also true for ODD and ADHD behavior and which differences between classrooms play a role.

In behavioral genetic studies, the absence of MI may have important consequences for heritability estimates. Absence of MI for an environmental factor, for example, gender of the teacher, could lead to differences in heritability estimates between groups (GxE interaction). Absence of MI for student’s gender may lead to what is known as scalar sex limitation, the effect of the genetic and environmental factors may, for example, be larger in boys than girls (Lubke et al. 2004; Neale et al. 2006). The short Conners’ Teacher Rating Scales—Revised (CTRS-R) is often filled out by teachers to assess ODD and ADHD behavior in a school setting (Conners et al. 1998). The scales of this instrument have been tested for MI in 7-year-old boys and girls (Derks et al. 2007a), showing no evidence for measurement bias regarding the gender of the student. However, the study did not take into account possible differences between male and female teachers in the perception of ODD and ADHD behavior nor did it evaluate MI at older ages. Therefore, the first objective of this study is to determine whether the scales of the CTRS-R, measuring ODD and ADHD behavior, are measurement invariant for gender of the student as well as gender of the teacher throughout primary school. When MI holds, the second objective of this study is to focus on GxE interaction, and investigate whether classroom sharing, gender of the student and gender of the teacher moderate the heritability of teacher-rated ODD and ADHD behavior.

## Methods

### Participants

The Netherlands Twin Register (NTR), established around 1987 by the Department of Biological Psychology at the VU University Amsterdam, registers approximately 40 % of all multiple births in the Netherlands. A survey about the development of the children is sent to the parents of the twins every 2 years until the twins are 12 years old (Boomsma et al. 2002, 2006; van Beijsterveldt et al. 2013). Since 1999, at approximately age 7, 9 and 12, when the twins attend primary school, parents are asked for their consent to approach the teacher(s) of their children with a survey. The survey sent to the primary school teachers includes items on background information of the teacher, functioning at school, educational achievement and the standardized questionnaires, the Teacher Report Form (TRF) (Achenbach 1991) and the short version of the Conners’ Teacher Ratings Scale—Revised (CTRS-R) (Conners 2001).

Since 2001 data collection has yielded surveys with information on gender of the teacher for 9,365, 8,775 and 6,649 7, 9 and 12-year-olds, respectively. We excluded children who had a disease or handicap that interfered severely with daily functioning (Age 7: N = 97; Age 9: N = 128; Age 12: N = 95) or attended specialized education, special schools are available for children with extra needs (Age 7: N = 109; Age 9: N = 237; Age 12: N = 226). Surveys were excluded if they were filled out by more than one teacher (Age 7: N = 431; Age 9: N = 259; Age 12: N = 83), filled out by someone other than the regular teacher (Age 7: N = 64; Age 9: N = 68; Age 12: N = 57), or if familiarity with the student was below average (Age 7: N = 53; Age 9: N = 62; Age 12: N = 34). This resulted in a total sample for the MI analyses of 8,611 surveys for 7-year-olds, 8,021 surveys for 9-year-olds and 5,954 surveys for 12-year-olds.

The sample for the GxE interaction analyses included complete phenotype data for most twin pairs (Age 7: N = 3,793; Age 9: N = 3,470; Age 12: N = 2,534). Incomplete data are due to only one of the teachers returning the survey. The sample consisted of 1,208, 1,102, and 762 twin pairs of opposite sex for respectively age 7, 9 and 12. For the same-sex twin pairs (Age 7: N = 2,585; Age 9: N = 2,368; Age 12: N = 1,772), determination of zygosity status was based on blood or DNA polymorphisms (Age 7: N = 224; Age 9: N = 331; Age 12: N = 393) or on the basis of parental report of items on resemblance in appearance and confusion of the twins by parents and others (Age 7: N = 2,321; Age 9: N = 1,987; Age 12: N = 1,356). This last method established zygosity with an accuracy of approximately 93 % (Rietveld et al. 2000). Zygosity was unavailable for some twins and these twin pairs were excluded from the analyses (Age 7: N = 40; Age 9: N = 50; Age 12: N = 23).

### Measures

The short Conners’ Teacher Rating Scale—Revised (CTRS-R) is a measurement instrument to asses ODD and ADHD behavior at school. Teachers had to indicate whether a child displayed a certain type of behavior currently or in the prior month. The short version of the CTRS-R consists of 28 items scored on a 4 point scale from 0 (not true or never) to 3 (completely true or very often) (Conners et al. 1998; Conners 2001). The CTRS-R includes 4 scales measuring Oppositional Behavior (OPP 5 items), Cognitive Problems/Inattention (ATT 5 items), Hyperactivity (HYP 7 items) and Attention Deficit Hyperactivity Disorder Index (ADHD 12 items). One item is included in both the HYP and ADHD scale (‘Easily excited, impulsive’). The item ‘Inattentive, gets distracted easily’ of the ADHD scale was excluded from the MI analyses as it was highly correlated with some of the other items, especially ‘Easily distracted or difficulty maintaining attention’ (Age 7: *r* = 0.812; Age 9: *r* = *0*.805; Age 12: *r* = 0.789) and ‘Short attention span’ (Age 7: *r* = 0.777; Age 9: *r* = 0.716; Age 12: *r* = 0.745). As a consequence, the more stringent MI models did not converge due to multicollinearity when including this item. For the GxE interaction analyses, a sum score of a scale was computed when there was at most one missing item (OPP, ATT and HYP) or at most two missing items (ADHD) for a scale. Missing items were imputed by the rounded averaged item score of the scale for that child. The sum scores of the scales showed an L-shaped distribution and therefore the data were square root transformed prior to the analyses.

### Statistical analyses

#### Measurement invariance

The factor structure of the four CTRS-R scales was investigated with exploratory factor analyses (EFA) with an Oblimin rotation. The number of latent factors was decided based on the scree plot and eigenvalues (larger than 1) of the factors. To test whether the scales of the CTRS-R were MI across student (‘boy’ or ‘girl’) gender and teacher (‘male’ or ‘female’) gender, multigroup (4 groups) confirmatory factor analyses (CFA) for ordinal item level data were carried out (Dolan 2000; Meredith 1993; Millsap and Yun-Tein 2004) using Mplus Version 6.1 (Muthén and Muthén 2010). With ordinal item level data an underlying continuously distributed liability is assumed and thresholds that categorize the disorder are estimated based on the response frequencies (Flora and Curran 2004). Because of the low frequencies of the most extreme response categories, the highest two response categories were combined. The EFA and CFA models were fitted with the Theta parameterization and the weighted least squares with mean variance adjusted (WLSMV) estimator. Correction for dependency of the observations due to family clustering was done by the ‘complex’ option. This ‘complex’ option computes the standard errors and a χ^2^ of model fit taking into account this dependency.

Different levels of MI were tested by constraining the model parameters step by step. The first level is configural invariance (configural MI), where the factor structure is the same across groups. Factor means are fixed to zero for identification purposes while factor variances, thresholds, loadings and residual variances of the continuous latent response variables are group specific. One of the factor loadings is constrained to be equal to 1 for scaling purposes. A stricter model is strong factorial invariance (strong MI), where differences in latent response means are the result of differences in the latent factor means. This model is tested by constraining both the factor loadings and thresholds to be equal across groups. The factor mean of the first group is fixed to zero and freely estimated in the other groups. The last model, strict factorial invariance (strict MI) implies that the differences in the latent response means reflect true differences in the latent factor means and variances. This is tested by constraining the factor loadings, thresholds and residual variances of the continuous latent response variables to be equal across all groups. The factor mean is still fixed to zero in the first group and freely estimated in the other groups (Dolan 2000; Mellenbergh 1989; Meredith 1993; Millsap and Yun-Tein 2004).

The root mean square error of approximation (RMSEA) and the comparative fit index (CFI) were chosen as indices of model fit. A RMSEA value smaller than 0.05 indicates a good fit as does a CFI value of 0.97 or higher (Schermelleh-Engel and Moosbrugger 2003). The difference in goodness of fit between the nested MI models in χ^2^ values between two nested models when using the WLMSV χ^2^ values is not distributed as a χ^2^ and as a consequence regular χ^2^ testing is not appropriate when using the WLSMV estimator (Muthén and Muthén 2010). Instead, the ‘difftest’ option in Mplus can be used to obtain a correct χ^2^ difference test by using the derivatives of the variables from both models. Due to the large sample sizes these χ^2^ difference tests models might reject a model on the basis of a significant χ^2^ difference even though the model actually fit. Interpreting the χ^2^ as a goodness-of-fit index has been suggested as an alternative for using the χ^2^ as a formal test statistic. Since there are no absolute standards, a ratio between 2 and 3 is proposed to be indicative of, respectively a good and an acceptable model fit (Schermelleh-Engel and Moosbrugger 2003). Therefore, a difference in χ^2^ of more than 3 times the difference in estimated parameters was interpreted as a worsening of the fit of the model. In addition, we looked at the parameter estimates and the magnitude of the modification indices to make reliable decisions on acceptance of MI.

#### Gene-environment interaction models

The contribution of genetic and environmental effects to the variance of the CTRS-R scales was estimated in a classical twin model (Boomsma et al. 2002; Plomin et al. 2008) in the R (R Core Team 2014) package OpenMx Version 3.1.0 (Boker et al. 2011, 2012) with maximum likelihood estimation. First, a saturated model was fitted to the data in which means, variances and covariances were estimated in the different zygosity-by-gender groups rated by same (ST) and different (DT) teachers. Mean and variance differences between children taught by male and female teachers, between boys and girls, between children sharing a classroom or in different classrooms and across zygosity were tested in the saturated model. It was tested whether the twin correlations could be equated between twins sharing a classroom and twins in different classrooms.

**Fig. 1:**
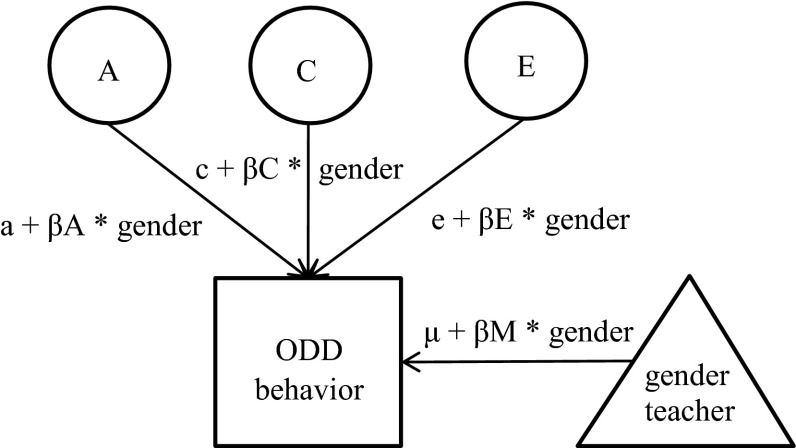
Gene-environment interaction (GxE) model with moderation by gender of the teacher

Next, GxE interaction models for gender of the student, classroom sharing and gender of the teacher were fitted to the data. GxE interaction was modelled by using multiple group designs for classroom sharing and gender of the student, and by a moderation model for teacher’s gender (Fig. 1) (Purcell 2002). The models included additive genetic effects (A), dominant genetic effects (D) (or common environmental effects (C), shared by twins) and unique environmental effects (E), not shared by twins. To correct for possible confounding by gene-environment correlation (rGE), means were allowed to be different between boys and girls, between twins rated by the same or different teachers and between children rated by male or female teachers (Purcell 2002). In the first models, differences in heritability between boys and girls were tested by constraining the estimates to be equal over gender of the student. Total variances between boys and girls were allowed to differ. Next, it was tested whether estimates could be constrained to be equal for twins rated by the same and by different teachers. Differences in genetic and environmental variance between the same and different teacher groups could be due to GxE interaction, but may also be the result of rater bias. Therefore, a correlated errors model was applied, which is an extension of the univariate twin model as it allows the unique environmental (E) effects to be correlated for twin pairs rated by the same teacher (Simonoff et al. 1998). In the last models, GxE interaction by gender of the teacher was tested by dropping from the model the moderation of the A, D (C) and E estimates by gender of the teacher.

Difference in goodness of fit of the nested models was assessed with a log-likelihood ratio test (LRT) which calculates the difference in −2log-likelihood (−2LL) between two models and evaluates this χ^2^-statistic with the difference in the number of estimated parameters between the models as degrees of freedom. A *p* value smaller than 0.01 was considered significant. Constraints were kept, when a more restrictive model did not significantly decrease the goodness of fit, as a more parsimonious model is preferred.

## Results

### Measurement invariance

MI of the four scales (OPP, ATT, HYP and ADHD) of the CTRS-R was tested across gender of the student (‘boy’ or ‘girl’) and gender of the teacher (‘male’ or ‘female’) at age 7 (Age: Mean = 7.44 and SD = 0.47), age 9 (Age: Mean = 9.92 and SD = 0.53) and age 12 (Age: Mean = 12.15 and SD = 0.30), resulting in a 4 group comparison. Information on the gender of the teacher was available for 8,611 7-year-olds (boy-male: N = 322; boy-female: N = 3,918; girl-male: N = 317; girl-female: 4,054), 8,021 9-year-olds (boy-male: N = 1,050; boy-female: N = 2,841; girl-male: N = 1,111; girl-female: N = 3,019) and 5,954 12-year-olds (boy-male: N = 1,332; boy-female: N = 1,503; girl-male: N = 1,381; girl-female: N = 1,738). Table 1 shows the frequencies of the item responses and the factor loadings of the items for all scales estimated from the EFA. Factor loadings were overall relatively high. On the basis of the scree plots and eigenvalues, a one-factor solution was chosen for OPP, ATT and HYP and a two-factor solution for ADHD (attention problems (AP) and hyperactivity/impulsivity (HI)) in all age groups (see Table 1).

**Table 1:** Frequencies of the item responses and factor loadings as estimated in the EFA

	Age 7					Age 9					Age 12				
	Frequencies of item responses			Factor loadings		Frequencies of item responses			Factor loadings		Frequencies of item responses			Factor loadings	
	0	1	2/3	1	2	0	1	2/3	1	2	0	1	2/3	1	2
Oppositional Behavior															
2 Defiant	0.828	0.141	0.031	0.917		0.797	0.167	0.036	0.914		0.781	0.181	0.038	0.915	
6 Defies	0.901	0.081	0.018	0.912		0.894	0.085	0.021	0.916		0.876	0.102	0.022	0.929	
10 Spiteful	0.959	0.034	0.007	0.777		0.931	0.059	0.010	0.832		0.937	0.054	0.009	0.803	
15 Argues	0.862	0.117	0.021	0.879		0.841	0.130	0.029	0.917		0.817	0.152	0.031	0.938	
20 Explosive	0.921	0.060	0.019	0.845		0.907	0.070	0.023	0.827		0.916	0.065	0.019	0.794	
Inattention/cognitive problems															
4 Forgets things	0.698	0.225	0.077	0.880		0.645	0.260	0.095	0.857		0.668	0.248	0.084	0.854	
8 Poor spelling	0.655	0.202	0.143	0.881		0.591	0.188	0.221	0.860		0.582	0.212	0.206	0.862	
13 Poor reading	0.696	0.153	0.151	0.844		0.728	0.134	0.137	0.799		0.786	0.117	0.097	0.824	
18 Lacks interest	0.842	0.120	0.039	0.698		0.797	0.159	0.045	0.595		0.778	0.170	0.052	0.617	
22 Poor arithmetic	0.748	0.171	0.081	0.770		0.695	0.175	0.130	0.743		0.702	0.175	0.123	0.748	
Hyperactivity															
3 Restless	0.680	0.221	0.099	0.766		0.706	0.209	0.085	0.743		0.766	0.176	0.058	0.757	
7 Always on the go	0.856	0.098	0.046	0.830		0.859	0.098	0.043	0.794		0.875	0.093	0.033	0.794	
11 Leaves seat	0.836	0.115	0.050	0.864		0.873	0.090	0.037	0.867		0.913	0.066	0.021	0.849	
17 Difficulty awaiting	0.703	0.204	0.093	0.828		0.756	0.167	0.077	0.843		0.804	0.140	0.056	0.851	
21 Runs about	0.937	0.047	0.016	0.876		0.950	0.038	0.012	0.878		0.964	0.028	0.008	0.884	
24 Difficulty playing	0.776	0.160	0.064	0.887		0.788	0.153	0.059	0.889		0.826	0.128	0.046	0.898	
27 Excitable	0.798	0.141	0.062	0.884		0.799	0.143	0.058	0.870		0.826	0.124	0.050	0.881	
ADHD Index															
Attention problems															
14 Short attention span	0.674	0.214	0.112	0.028	0.938	0.687	0.203	0.110	0.076	0.897	0.726	0.194	0.079	0.008	0.944
16 Only attention for own interests	0.785	0.160	0.054	0.202	0.585	0.757	0.180	0.063	0.204	0.583	0.750	0.184	0.066	0.193	0.609
19 Distractible	0.645	0.231	0.123	0.102	0.887	0.649	0.226	0.124	0.164	0.832	0.687	0.222	0.091	0.091	0.879
25 Fails to finish	0.792	0.164	0.044	−0.045	0.908	0.797	0.163	0.040	−0.065	0.928	0.824	0.142	0.033	−0.061	0.929
26 Not following instructions	0.875	0.088	0.037	−0.080	0.925	0.883	0.083	0.034	−0.094	0.949	0.895	0.080	0.024	−0.035	0.913
Hyperactivity															
5 Disturbs other children	0.709	0.228	0.063	0.855	0.023	0.696	0.237	0.067	0.854	0.026	0.730	0.210	0.060	0.840	0.051
9 Cannot remain still	0.779	0.160	0.062	0.848	0.106	0.786	0.160	0.054	0.808	0.150	0.825	0.136	0.039	0.839	0.106
12 Fidgets	0.709	0.197	0.094	0.676	0.174	0.754	0.168	0.078	0.596	0.243	0.825	0.132	0.044	0.658	0.162
23 Interrupts	0.750	0.191	0.059	0.920	−0.076	0.754	0.187	0.059	0.910	−0.070	0.797	0.160	0.043	0.900	−0.032
27 Excitable	0.798	0.141	0.062	0.893	−0.057	0.799	0.143	0.058	0.909	−0.080	0.826	0.124	0.050	0.911	−0.078
28 Restless	0.814	0.129	0.056	0.944	0.004	0.821	0.127	0.053	0.914	0.035	0.850	0.116	0.034	0.958	−0.024

Results for the tests of the three levels of MI are reported in Table S1. For OPP, HYP and ADHD the configural, strong and strict invariance models all showed an acceptable to good fit, based on the RMSEA and CFI, for all age groups. Differences in χ^2^ between the models with increasing equality constraints were rather small and, for the strong MI level, did not exceed more than three times the number of degrees of freedom. However, for the strict MI level, the difference in a χ^2^ for OPP at age 9 and HYP at age 7 and 12 was somewhat larger than this criterion, but these differences were accompanied by minor changes in RMSEA and CFI. Inspection of the modification indices revealed that they were larger for female teachers compared to male teachers for both boys and girls. Taken together, we could accept MI for the scales OPP, HYP and ADHD, for all ages, with respect to gender of the student and, more tentatively, for gender of the teacher. The fit of the MI models was acceptable to mediocre for ATT in 7-year-olds while the fit of the models was unacceptable for 9 and 12-year-olds. Even the models without constraints on the factor structure did not fit the data very well. Increasing MI levels led to a large decrease in model fit for all ages. Therefore, we could not accept MI across gender of the student and teacher for the ATT scale.

### Gene x environment interaction models

Table 2 gives the means and standard deviations of the measurement invariant CTRS-R scales for boys and girls with the same or different male or female teachers across the three age groups. The saturated models were used to test for mean and variance differences across these groups. For OPP, there were mean and variance differences between boys and girls at all ages and variance differences across zygosity at age 7, between children sharing a classroom and children in different classrooms at age 12 and between children with the same or different male or female teachers at age 12. For HYP, there were mean and variance differences between boys and girls at all ages, mean differences across zygosity and between children sharing a classroom and children in different classrooms at age 7 and variance differences between children sharing a classroom and children in different classrooms at age 12. For ADHD, there were mean and variance differences between boys and girls at all ages and mean differences between children sharing a classroom and children in different classrooms at all ages.

**Table 2:** Means and standard deviations of the untransformed sum scores of the CTRS-R scales at age 7, 9 and 12

	Male teacher								Female teacher							
	Same teacher				Different teacher				Same teacher				Different teacher			
	Boys		Girls		Boys		Girls		Boys		Girls		Boys		Girls	
	N	Mean (SD)	N	Mean (SD)	N	Mean (SD)	N	Mean (SD)	N	Mean (SD)	N	Mean (SD)	N	Mean (SD)	N	Mean (SD)
Oppositional Behavior																
Age 7	167	0.8 (1.7)	170	0.5 (1.3)	109	0.7 (1.6)	107	0.3 (1.1)	1,910	0.8 (1.8)	2,091	0.4 (1.1)	1,489	0.9 (1.8)	1,468	0.4 (1.2)
Age 9	557	1.0 (1.9)	594	0.5 (1.2)	347	0.9 (1.9)	349	0.6 (1.6)	1,401	1.1 (2.0)	1,576	0.5 (1.3)	1,002	1.0 (2.1)	1,039	0.5 (1.4)
Age 12	748	1.0 (2.0)	814	0.5 (1.1)	381	1.0 (1.9)	365	0.6 (1.4)	805	1.2 (2.1)	959	0.4 (1.2)	442	1.0 (2.2)	497	0.7 (1.6)
Hyperactivity																
Age 7	167	2.5 (3.6)	170	1.5 (2.7)	108	2.3 (3.0)	106	0.9 (2.1)	1,907	2.7 (3.9)	2,093	1.1 (2.2)	1,486	2.9 (3.9)	1,469	1.2 (2.3)
Age 9	556	2.3 (3.4)	592	1.0 (1.9)	347	2.3 (3.4)	351	1.1 (2.2)	1,399	2.5 (3.6)	1,578	0.9 (1.8)	1,000	2.7 (3.8)	1,038	1.0 (2.3)
Age 12	752	1.8 (3.0)	815	0.8 (1.8)	381	1.8 (2.8)	366	0.9 (1.9)	804	2.0 (3.2)	959	0.6 (1.5)	442	2.2 (3.6)	496	0.9 (2.1)
ADHD index																
Age 7	167	5.3 (6.6)	170	3.4 (5.4)	108	4.6 (5.0)	107	2.9 (4.4)	1,906	5.3 (6.6)	2,091	2.9 (4.6)	1,485	6.2 (7.1)	1,469	3.3 (4.9)
Age 9	553	5.1 (6.4)	589	2.9 (4.6)	348	5.5 (6.9)	351	3.1 (4.6)	139	5.6 (6.7)	1,578	2.6 (4.2)	999	6.3 (7.0)	1,039	3.0 (4.7)
Age 12	750	4.5 (6.0)	815	2.3 (3.7)	381	4.7 (5.6)	366	2.5 (3.9)	804	4.9 (6.2)	960	1.9 (3.6)	439	5.6 (6.9)	495	2.6 (4.3)

*N* number of observations, *SD* standard deviation

**Fig. 2:**
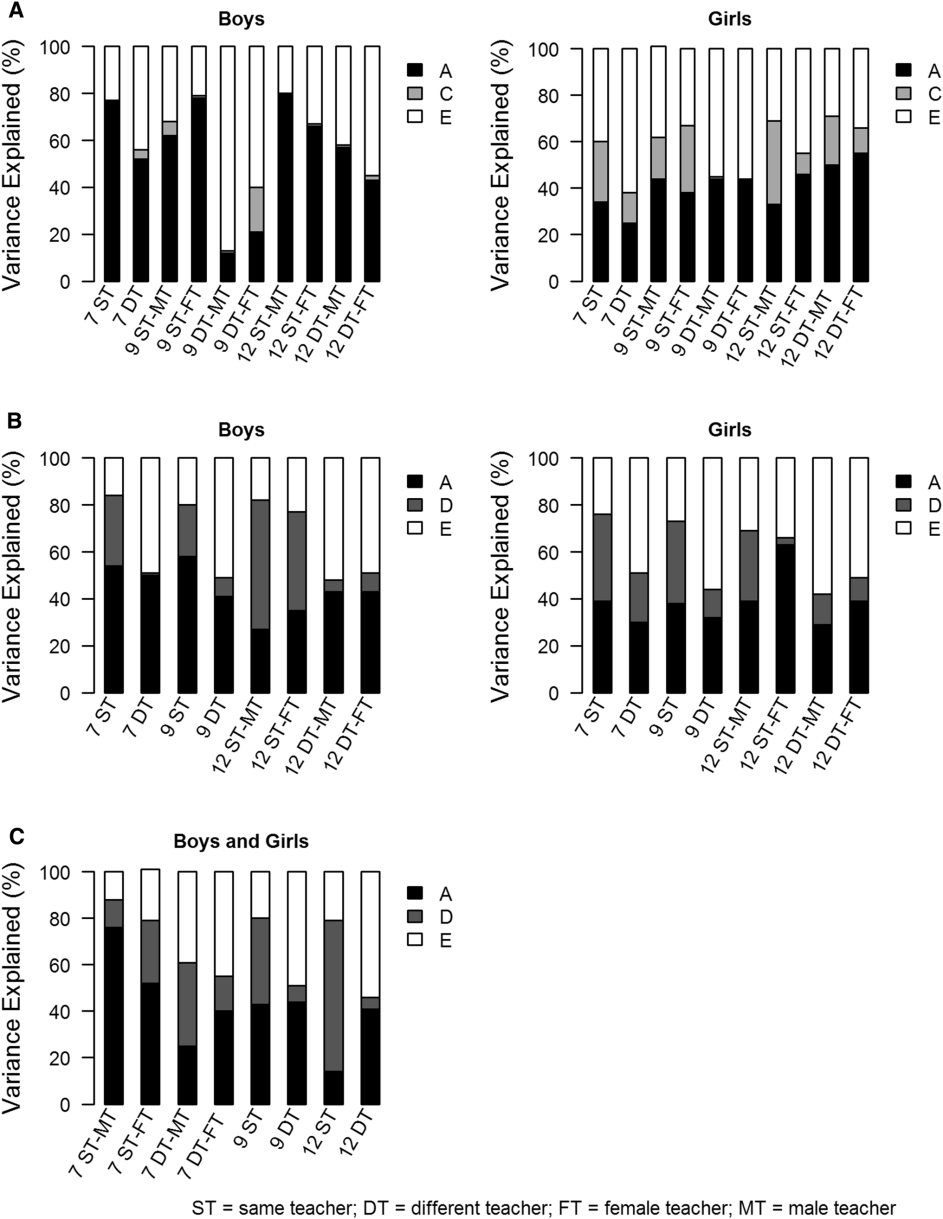
The relative contribution of the additive genetic, dominant genetic, common environmental and unique environmental effects for the most parsimonious and best fitting models for Oppositional Behavior (**a**), Hyperactivity (**b**) and Attention Deficit Hyperactivity Disorder Index (**c**)

Twin correlations for each gender by zygosity group rated by the same teacher or by different teachers are given in Table 3. For all scales, MZ correlations were higher, sometimes more than twice as high, than DZ correlations, suggesting additive (and in some cases dominant) genetic effects. Only for the OPP scale were DZ correlations larger than half the MZ correlations, suggesting common environmental effects. The GxE interaction model fitting results are reported in the online supplementary materials for the OPP (Table S2), HYP (Table S3) and ADHD (Table S4) scales of the CTRS-R. The standardized estimates (Table 4) and the contribution of the variance components (Fig. 2) are given for the most parsimonious and best fitting models.

**Table 3:** Twin correlations for the CTRS-R scales rated by the same teacher or different teachers at age 7, 9 and 12

	Oppositional Behavior		Hyperactivity		ADHD index	
	ST	DT	ST	DT	ST	DT
Age 7						
MZm	0.772	0.495	0.842	0.479	0.820	0.555
DZm	0.360	0.280	0.347	0.289	0.437	0.292
MZf	0.617	0.394	0.749	0.492	0.770	0.514
DZf	0.404	0.233	0.310	0.211	0.342	0.217
DOS	0.294	0.112	0.301	0.176	0.339	0.250
Age 9						
MZm	0.763	0.334	0.790	0.465	0.792	0.447
DZm	0.405	0.211	0.342	0.208	0.353	0.296
MZf	0.635	0.442	0.712	0.407	0.793	0.497
DZf	0.498	0.081	0.302	0.145	0.379	0.270
DOS	0.244	0.133	0.296	0.242	0.327	0.254
Age 12						
MZm	0.719	0.518	0.792	0.434	0.818	0.546
DZm	0.350	0.282	0.297	0.310	0.283	0.301
MZf	0.606	0.500	0.681	0.361	0.751	0.414
DZf	0.338	0.297	0.315	0.282	0.276	0.245
DOS	0.232	0.185	0.234	0.205	0.265	0.233

*ST* same teacher, *DT* different teacher; *MZm* monozygotic boys, *DZm* dizygotic boys, *MZf* monozygotic girls, *DZf* dizygotic girls, *DOS* dizygotic of opposite sex

**Table 4:** Standardized estimates [95% Confidence intervals] of the total genetic (G), additive genetic (A), dominant genetic (D), common environmental (C) and unique environmental (E) effects on the four CTRS-R scales for 7, 9 and 12-year-olds in the best-fitting models

	Same teacher					Different teacher				
	G	A	C	D	E	G	A	C	D	E
Oppositional Behavior										
Age 7										
Boys	.77 [0.71–.80]	.77 [0.71–.80]	.00 [0.00–.05]		.23 [0.20–.27]	.52 [0.32–.62]	.52 [0.32–.62]	.04 [0.00–.20]		.45 [0.37–.53]
Girls	.34 [0.15–.54]	.34 [0.15–.54]	.26 [0.07–.43]		.40 [0.35–.46]	.25 [0.00–.42]	.25 [0.00–.42]	.13 [0.00–.34]		.62 [0.53–.72]
Age 9										
MT										
Boys	.62 [0.41–.74]	.62 [0.41–.74]	.06 [0.00–.25]		.32 [0.24–.41]	.12 [0.00–.29]	.12 [0.00–.29]	.01 [0.00–.13]		.87 [0.71–.99]
Girls	.44 [0.20–.66]	.44 [0.20–.66]	.18 [0.00–.38]		.39 [0.30–.50]	.44 [0.30–.56]	.44 [0.30–.56]	.01 [0.00–.09]		.56 [0.43–.70]
FT										
Boys	.78 [0.69–.82]	.78 [0.69–.82]	.01 [0.00–.09]		.21 [0.18–.25]	.21 [0.00–.25]	.21 [0.00–.25]	.19 [0.04–.36]		.60 [0.49–.72]
Girls	.38 [0.21–.55]	.38 [0.21–.55]	.29 [0.13–.44]		.34 [0.13–.44]	.44 [0.31–.54]	.44 [0.31–.54]	.00 [0.00–.08]		.56 [0.46–.69]
Age 12										
MT										
Boys	.80 [0.72–.84]	.80 [0.72–.84]	.00 [0.00–.07]		.20 [0.16–.25]	.57 [0.34–.69]	.57 [0.34–.69]	.01 [0.00–.18]		.42 [0.31–.55]
Girls	.33 [0.13–.56]	.33 [0.13–.56]	.36 [0.15–.54]		.31 [0.25–.38]	.50 [0.27–.70]	.50 [0.27–.70]	.21 [0.04–.43]		.29 [0.19–.42]
FT										
Boys	.66 [0.53–.73]	.66 [0.53–.73]	.01 [0.00–.20]		.33 [0.26–.41]	.43 [0.22–.55]	.43 [0.22–.55]	.02 [0.00–.20]		.55 [0.44–.68]
Girls	.46 [0.27–.60]	.46 [0.27–.60]	.09 [0.00–.25]		.44 [0.36–.54]	.55 [0.35–.69]	.55 [0.35–.69]	.11 [0.00–.30]		.34 [0.25–.46]
Hyperactivity										
Age 7										
Boys	.84 [0.81–.86]	.54 [0.20–.86]		.30 [0.00–.64]	.16 [0.14–.19]	.51 [0.43–.59]	.50 [0.17–.59]		.01 [0.00–.36]	.49 [0.41–.57]
Girls	.75 [0.72–.78]	.39 [0.16–.78]		.37 [0.00–.60]	.25 [0.21–.28]	.51 [0.42–.58]	.30 [0.04–.55]		.21 [0.00–.49]	.49 [0.42–.58]
Age 9										
Boys	.80 [0.76–.83]	.58 [0.20–.82]		.22 [0.00–.60]	.20 [0.17–.24]	.49 [0.39–.58]	.41 [0.10–.57]		.08 [0.00–.42]	.51 [0.42–.61]
Girls	.72 [0.68–.76]	.38 [0.15–.75]		.35 [0.00–.58]	.28 [0.24–.32]	.44 [0.33–.53]	.32 [0.06–.51]		.12 [0.00–.41]	.56 [0.47–.67]
Age 12										
MT										
Boys	.82 [0.77–.86]	.27 [0.00–.62]		.55 [0.20–.82]	.18 [0.14–.23]	.48 [0.34–.60]	.43 [0.09–.59]		.05 [0.00–.40]	.52 [0.40–.66]
Girls	.68 [0.61–.74]	.39 [0.00–.70]		.30 [0.00–.69]	.32 [0.26–.39]	.43 [0.24–.57]	.29 [0.03–.50]		.13 [0.00–.43]	.57 [0.43–.76]
FT										
Boys	.76 [0.70–.81]	.35 [0.03–.68]		.42 [0.09–.74]	.24 [0.19–.30]	.51 [0.37–.62]	.43 [0.11–.60]		.08 [0.00–.41]	.49 [0.38–.63]
Girls	.66 [0.58–.73]	.63 [0.34–.72]		.03 [0.00–.33]	.34 [0.27–.42]	.49 [0.27–.42]	.39 [0.11–.58]		.10 [0.00–.38]	.51 [0.39–.65]
ADHD Index										
Age 7										
MT	.88 [0.83–.92]	.76 [0.35–.90]		.12 [0.00–.53]	.12 [0.08–.17]	.61 [0.48–.72]	.25 [0.07–.63]		.36 [0.00–.62]	.39 [0.28–.52]
FT	.78 [0.76–.81]	.52 [0.34–.69]		.27 [0.10–.44]	.22 [0.19–.24]	.55 [0.50–.60]	.40 [0.18–.56]		.15 [0.00–.38]	.45 [0.40–.50]
Age 9	.80 [0.77–.82]	.43 [0.25–.60]		.37 [0.20–.55]	.20 [0.18–.23]	.50 [0.43–.57]	.44 [0.21–.55]		.07 [0.00–.31]	.50 [0.43–.57]
Age 12	.79 [0.76–.81]	.14 [0.00–.34]		.65 [0.44–.80]	.21 [0.19–.24]	.46 [0.38–.53]	.41 [0.11–.52]		.05 [0.00–.38]	.54 [0.46–.62]

*G* genetic effects (summation of additive and dominant genetic effects),* A* additive genetic effects,* C* common environmental effects,* E* unique environmental effects,* MT* male teacher,* FT* female teacher

#### Classroom sharing

Correlations between twins rated by the same teacher could not be constrained to be equal to correlations between twins with different teachers. Constraining the variance components to be equal across same and different teachers also resulted in a significant deterioration of the model fit. A model with correlated errors was fitted to the data to check whether the differences between the same teacher and different teacher groups could be explained by rater bias. For none of the scales did the correlated errors model provide a better fit. In general, the proportion of the variance explained by genetic effects (heritability) was higher, at all ages, for children taught by the same teacher (ST) than for children rated by different teachers (DT) for OPP in boys (ST 62–80 %; DT 12–57 %) and girls (ST 33–46 %; DT 25–55 %), HYP in boys (ST 76–84 %; DT 48–51 %) and girls (ST 66–75 %; DT 43–51 %) and ADHD (ST 78–88 %; DT 46–61 %).

#### Gender of the student

For the scales OPP and HYP, the contribution of the variance components differed between boys and girls at all ages, while this was not the case for the ADHD scale. Heritability of OPP was higher for boys (ST 62–80 %; DT 12–57 %) than girls (ST 33–46 %; DT 25–55 %). The influence of common environmental effects was, at most ages, negligible in boys (ST 0–6 %; DT 1–19 %) while it had some influence in girls (ST 9–36 %; DT 0–21 %). Heritability of HYP was slightly higher for boys (ST 76–84 %; DT 48–51 %) than girls (ST 66–75 %; DT 43–51 %). Differences between boys and girls on this scale could mainly be attributed to differences in the influence of dominant genetic effects.

#### Gender of the teacher

Moderation by gender of the teacher was significant for OPP at age 9 and 12, HYP at age 12 and ADHD at age 7. For OPP at age 9, the relative influence of genetic effects was larger in boys with female teachers (ST 78 %; DT 21 %) than with male teachers (ST 62 %; DT 12 %) while it was somewhat larger for girls with male teachers (ST 44 %; DT 44 %) compared to with female teachers (ST 38 %; DT 44 %). For OPP at age 12, the opposite was true; heritability was larger in boys with male teachers (ST 80 %; DT 57 %) than with female teachers (ST 66 %; DT 43 %) while heritability was somewhat larger when girls were taught by a female teacher (ST 46 %; DT 55 %) compared to when they were taught by a male teacher (ST 33 %; DT 50 %). For HYP at age 12, heritability was almost equal in boys and girls with male and female teachers, but the extent to which dominant genetic effects played a role differed across gender of the teacher. For ADHD at age 7, heritability was larger for children with male teachers (ST 88 %; DT 61 %) compared to with female teachers (ST 78 %; DT 55 %).

## Discussion

Three (OPP, HYP and ADHD) of the four scales of the short Conners’ Teacher Ratings Scale—Revised (CTRS-R) (Conners 2001), used in a school setting to assess ODD and ADHD behavior, were measurement invariant across gender of the student and teacher. This means that gender differences in means and variances may be interpreted as reflecting true differences on the underlying disorder. In contrast, MI did not hold for the Inattention/Cognitive Problems (ATT) scale. Explanations for the absence of MI could be the low factor loadings and the moderate test–retest reliability of this scale. Problems with the item content have been previously suggested (Conners et al. 1998). In our sample, the internal reliability of the Inattention/Cognitive Problems scale of the short CTRS-R ranged from 0.78 to 0.82. The results of the MI analyses strongly question the reliability of this scale and its use in clinical practice. Revision of this scale is recommended as the ratings might reflect a bias instead of true differences.

Heritability of ODD and ADHD behavior, measured with the OPP, HYP and ADHD scales of the CTRS-R is substantial. Common environmental effects had some influence on ODD behavior while dominant genetic effects had an influence on ADHD behavior. The finding of common environmental effects is consistent with earlier studies of ODD behavior using parental ratings (Burt et al. 2001; Tuvblad et al. 2009). The influence is larger in girls which may be explained by the fact that girls appear to be more sensitive to reprimands from the teacher than boys. Earlier research already concluded that girls more often feel the pressure from peers or others to behave prosocially (Roberts and Strayer 1996). Girls might be more inclined to adapt their behavior when they are called upon by the teacher. In younger girls the common environment also has an influence when they do not share a classroom. Factors in the home environment that have been proposed to have an influence on ODD behavior are, for example, parental discipline and parental involvement (Frick et al. 1992) and the influence of these factors could depend on the gender of a child and decrease when a child grows older. The finding of dominant genetic effects for ADHD behavior, especially in children sharing a classroom, could also be due to rater contrast effects. Only when one teacher rates both children of a twin pair can the behavior of the children be contrasted and result in negative interaction effects. A higher rating for ADHD behavior in one of the children of a twin pair could lead to a lower rating for ADHD behavior in the co-twin. However, the variance in ADHD behavior is not significantly smaller in MZ twin pairs compared to DZ twin pairs, which disconfirms the presence of this type of rater bias. This is in accordance with the results of a study looking into mother and teacher ratings of hyperactivity. A contrast effect was found for the maternal ratings while the teacher ratings did not show this form of rater bias (Simonoff et al. 1998).

Heritability estimates for ADHD behavior are comparable to those found in studies taking differences between same and different teachers into account. For example, Merwood et al. (2013) also found differences in heritability between 12-year-old children sharing a classroom (76 %) and not sharing a classroom (49 %). One study included only twin pairs sharing a classroom and observed a heritability of 74 % (Hartman et al. 2007) while another included only twins not sharing a classroom and estimated a heritability of 46 % (Towers et al. 2000). GxE interaction was the most plausible explanation for internalizing and externalizing problems, assessed with the Teacher Report Form, in 7 to 12-year-old twin pairs of which approximately 60 % shared a classroom (Lamb et al. 2012). Other studies looking into GxE interaction for ADHD in 11–12-year-olds (Merwood et al. 2013), and hyperactivity in 7-year olds (Saudino et al. 2005) also observed that heritability was larger when children shared a classroom. On the other hand, a study in 7-year-olds did not observe a difference between children sharing a classroom and children in different classrooms in the heritability of ODD and ADHD behavior (Derks et al. 2007a), but it could be that this study did not have enough power to detect these differences in the heritability (Derks et al. 2004).

Studies towards the heritability of teacher-rated ODD behavior are scarce. The findings of gender differences and common environmental effects were in accordance with the results of a study by Hudziak et al. (2005) that was based on a subsample of the present study. In contrast with current findings, none of the heritability estimates of the maternal-rated ODD behavior differed between boys and girls (Dick et al. 2005; Tuvblad et al. 2009). The differences between parent and teacher ratings of ODD behavior could be due to the fact that children can express different behavior in the classroom than they do at home. The OPP scale of the CTRS-R takes these differences into account by including different items for the teacher survey. A study observed that, although parents rated children rather similar over time, teachers with different teaching styles rated the same children very different across grades, suggesting that behavior differed in response to different teaching styles (Vitaro et al. 1995). Another explanation is that teachers have highly informed views on general childhood behavior for both boys and girls and are better able to assess which behavior is normative for a child of a certain age and gender.

Heritability of ODD and ADHD behavior was larger in children who shared a classroom compared to those who did not. The correlated errors model did not provide a better explanation for the differences in correlations between children rated by the same and different teachers, excluding teacher bias as an explanation, and therefore these findings are in line with GxE interaction for classroom sharing. In general, the heritability of ODD and ADHD behavior was lower in children not sharing a classroom leading to a larger impact of the environment which suggests that different behavior is elicited by different classroom environments. The children are taught by different teachers, with different rules and teaching methods and have different peers. All these factors could contribute to differences between children. For example, how teachers handle disruptive behavior is related to the behavior of a child (Rydell and Henricsson 2004). The unique environmental variance also contains measurement error which might be increased when different teachers rate the two children of a twin pair as rater variance ends up in the measurement error (Hoyt 2000). An important question is which differences between classroom environments play a role. Peer problems are related to ODD and ADHD behavior (Paap et al. 2013). Genetic variance in childhood aggression is moderated by peer victimization and might also moderate the heritability of ODD and ADHD (Brendgen et al. 2008). A study towards differences between monozygotic twins in their perception of the classroom environment identified, for example, the perception of a student about the relationship with the teacher as a unique environmental factor that differed between the genetically identical twins and was linked to hyperactivity as rated by the teacher (Somersalo et al. 2002).

For one teacher characteristic, gender, we investigated whether it moderated genetic effects on behavior in the classroom. The expression of a child’s genetic vulnerability for displaying ODD and ADHD behavior at school depended in some cases on the gender of the teacher. The direction of the difference in heritability may provide an indication for one of two hypotheses. Male teachers and female teachers could provide a different learning and classroom environment with regard to, for example, structure and rules. The bioecological model (Bronfenbrenner and Ceci 1994) predicts that the heritability of a phenotype will be lower in an adverse environment because risk environments will prevent the amplification of underlying genetic differences between children while the diathesis-stress model suggests that heritability will be higher in an adverse environment due to the expression of a genetic vulnerability that is triggered by a risk environment (Rende and Plomin 1992). A same-gender teacher might be seen as a supportive environment as it is suggested to have a positive influence on the behavior and educational achievement of a child (Carrington et al. 2008). According to the bioecological model, genetic variation will be higher when children are taught by a same-gender teacher while the diasthesis-stress model predicts that heritability will be lower. However, in our study, the direction of the effects of gender of the teacher was not consistent which makes interpreting the GxE interaction findings difficult.

To summarize, three of the four scales of the short CTRS-R measuring teacher-rated ODD and ADHD behavior in 7, 9 and 12-year-olds were measurement invariant for student gender and teacher gender. Revision of the fourth scale (ATT) is highly recommended in order to be useable in clinical practice. The heritability of ODD and ADHD behavior was lower for children in different classrooms compared to children sharing a classroom, suggesting that different behavior is elicited by different classroom environments. Apparently, teachers, the classroom and/or peers are important environmental factors that influence the expression of ODD and ADHD behavior in primary school. The direction of the moderation of the heritability of ODD and ADHD behavior by gender of the teacher was not consistent, which makes interpretation difficult. Finding environmental factors with a moderating influence on the heritability ODD and ADHD might help improve learning environments at school to prevent manifestation of ODD and ADHD behavior in children with an increased genetic vulnerability for these disorders.

## Electronic supplementary material

Below is the link to the electronic supplementary material.

Supplementary material 1 (DOCX 46 kb)
