# Supplementary material for: Childhood ODD and ADHD Behavior: The Effect of Classroom Sharing, Gender, Teacher Gender and Their Interactions
Source: Behav Genet. 2015 Feb 25;45(4):394–408. doi: 10.1007/s10519-015-9712-z (PMC4458263; doi:10.1007/s10519-015-9712-z)
Supplement: Supplementary file 1 — Supplementary material 1 (DOCX 46 kb) [file 10519_2015_9712_MOESM1_ESM.docx]

**Table S1** Model fitting results for measurement invariance tested in three age groups across gender of the teacher and gender of the student

|  | | | | **N** | | **ep** | | **RMSEA** | **χ^2^** | **CFI** | **χ^2^**  **Difference Test** | **df** | **p** |
| --- | --- | --- | --- | --- | --- | --- | --- | --- | --- | --- | --- | --- | --- |
| **Oppositional Behavior** | | | | | | |  | |  |  |  |  |  |
| Age 7 | | EFA | 8552 | | 60 | | .058 | | 150.195 | .994 |  |  |  |
|  | | Configural | 8552 | | 60 | | .060 | | 173.850 | .993 |  |  |  |
|  | | Strong | 8552 | | 36 | | .034 | | 188.452 | .994 | 50.395 | 24 | .001 |
|  | | Strict | 8552 | | 21 | | .039 | | 202.633 | .994 | 25.778 | 15 | .040 |
| Age 9 | | EFA | 7962 | | 60 | | .073 | | 215.595 | .993 |  |  |  |
|  | | Configural | 7962 | | 60 | | .074 | | 237.804 | .993 |  |  |  |
|  | | Strong | 7962 | | 36 | | .044 | | 214.997 | .994 | 33.557 | 24 | .093 |
|  | | Strict | 7962 | | 21 | | .042 | | 263.845 | .993 | 58.267 | 15 | <.001 |
| Age 12 | | EFA | 5904 | | 60 | | .065 | | 130.095 | .996 |  |  |  |
|  | | Configural | 5904 | | 60 | | .065 | | 143.429 | .996 |  |  |  |
|  | | Strong | 5904 | | 36 | | .041 | | 152.748 | .996 | 45.131 | 24 | .006 |
|  | | Strict | 5904 | | 21 | | .037 | | 180.625 | .996 | 33.185 | 15 | .004 |
| **Cognitive Problems/**  **Inattention** | | | | | | | | |  |  |  |  |  |
| Age 7 | | EFA | 8551 | | 60 | | .094 | | 382.373 | .986 |  |  |  |
|  | | Configural | 8551 | | 60 | | .091 | | 376.516 | .987 |  |  |  |
|  | | Strong | 8551 | | 36 | | .079 | | 633.634 | .979 | 303.322 | 24 | <.001 |
|  | | Strict | 8551 | | 21 | | .073 | | 723.741 | .976 | 126.082 | 15 | <.001 |
| Age 9 | | EFA | 7963 | | 60 | | .145 | | 840.426 | .956 |  |  |  |
|  | | Configural | 7963 | | 60 | | .140 | | 799.807 | .963 |  |  |  |
|  | | Strong | 7963 | | 36 | | .130 | | 1528.966 | .930 | 765.792 | 24 | <.001 |
|  | | Strict | 7963 | | 21 | | .119 | | 1721.781 | .921 | 250.020 | 15 | <.001 |
| Age 12 | | EFA | 5904 | | 60 | | .147 | | 645.088 | .956 |  |  |  |
|  | | Configural | 5904 | | 60 | | .147 | | 660.227 | .961 |  |  |  |
|  | | Strong | 5904 | | 36 | | .131 | | 1150.344 | .932 | 530.606 | 24 | <.001 |
|  | | Strict | 5904 | | 21 | | .119 | | 1291.816 | .925 | 166.737 | 15 | <.001 |
| **Hyperactivity** | | |  | |  | |  | |  |  |  |  |  |
| Age 7 | | EFA | 8552 | | 84 | | .044 | | 242.830 | .995 |  |  |  |
|  | | Configural | 8552 | | 84 | | .041 | | 261.458 | .995 |  |  |  |
|  | | Strong | 8552 | | 48 | | .035 | | 329.143 | .994 | 100.176 | 36 | <.001 |
|  | | Strict | 8552 | | 27 | | .033 | | 383.403 | .993 | 77.061 | 21 | <.001 |
| Age 9 | | EFA | 7959 | | 84 | | .043 | | 221.821 | .994 |  |  |  |
|  | | Configural | 7959 | | 84 | | .043 | | 267.452 | .994 |  |  |  |
|  | | Strong | 7959 | | 48 | | .033 | | 288.498 | .994 | 75.832 | 36 | <.001 |
|  | | Strict | 7959 | | 27 | | .031 | | 330.362 | .993 | 59.778 | 21 | <.001 |
| Age 12 | | EFA | 5904 | | 84 | | .038 | | 134.893 | .995 |  |  |  |
|  | | Configural | 5904 | | 84 | | .041 | | 194.261 | .993 |  |  |  |
|  | | Strong | 5904 | | 48 | | .029 | | 208.982 | .994 | 50.365 | 36 | .056 |
|  | | Strict | 5904 | | 27 | | .032 | | 281.340 | .992 | 75.149 | 21 | <.001 |
| **ADHD Index** | | |  | |  | |  | |  |  |  |  |  |
| Age 7 | EFA | | 8552 | | 136 | | .086 | | 2205.268 | .984 |  |  |  |
|  | Configural | | 8552 | | 136 | | .070 | | 1983.366 | .986 |  |  |  |
|  | Strong | | 8552 | | 82 | | .060 | | 1948.785 | .987 | 100.227 | 54 | <.001 |
|  | Strict | | 8552 | | 49 | | .050 | | 1661.353 | .989 | 47.255 | 33 | .052 |
| Age 9 | EFA | | 7961 | | 136 | | .082 | | 1868.673 | .985 |  |  |  |
|  | Configural | | 7961 | | 136 | | .073 | | 1979.756 | .984 |  |  |  |
|  | Strong | | 7961 | | 82 | | .063 | | 2012.996 | .984 | 155.307 | 54 | <.001 |
|  | Strict | | 7961 | | 49 | | .054 | | 1757.824 | .986 | 60.369 | 33 | .003 |
| Age 12 | EFA | | 5904 | | 136 | | .078 | | 1270.317 | .985 |  |  |  |
|  | Configural | | 5904 | | 136 | | .064 | | 1214.061 | .986 |  |  |  |
|  | Strong | | 5904 | | 82 | | .054 | | 1201.933 | .987 | 81.171 | 54 | .010 |
|  | Strict | | 5904 | | 49 | | .048 | | 1143.753 | .988 | 90.742 | 33 | <.001 |

^N = number of observations; ep = estimated parameters; RMSEA = root mean square error of approximation; Χ2 = chi square;^

^CFI = comparative fit index; df = degrees of freedom; EFA = exploratory factor analysis^

**Table S2** Genetic modeling results for the oppositional behavior (OPP) scale

|  | **ep** | **-2ll** | **df** | **model** | **χ2** | **Δdf** | **p** |
| --- | --- | --- | --- | --- | --- | --- | --- |
| **Age 7** |  |  |  |  |  |  |  |
| 0 Saturated | 52 | 14503.83 | 7379 | - | - | - | - |
| 1 Saturated: ST = DT | 37 | 14614.79 | 7394 | 0 | 110.96 | 15 | <.001 |
| 2 ACE | 23 | 14583.00 | 7408 | 0 | 79.17 | 29 | <.001 |
| 3 ACE: Boys = Girls | 15 | 14673.59 | 7416 | 2 | 90.58 | 8 | <.001 |
| 4 ACE: ST = DT | 17 | 14656.08 | 7414 | 2 | 73.08 | 6 | <.001 |
| 5 ACE: Correlated Errors | 18 | 14592.64 | 7413 | 0 | 87.79 | 33 | <.001 |
| *6 ACE: FT = MT* | *17* | *14587.70* | *7414* | *2* | *4.70* | *6* | *.583* |
| **Age 9** |  |  |  |  |  |  |  |
| 0 Saturated | 52 | 14271.56 | 6713 | - | - | - | - |
| 1 Saturated: ST = DT | 37 | 14417.89 | 6728 | 0 | 146.33 | 15 | <.001 |
| *2 ACE* | *23* | *14302.35* | *6742* | *0* | *30.79* | *29* | *.375* |
| 3 ACE: Boys = Girls | 15 | 14385.60 | 6750 | 2 | 83.25 | 8 | <.001 |
| 4 ACE: ST = DT | 17 | 14428.55 | 6748 | 2 | 126.19 | 6 | <.001 |
| 5 ACE: Correlated Errors | 18 | 14349.08 | 6747 | 0 | 75.60 | 33 | <.001 |
| 6 ACE: FT = MT | 17 | 14322.82 | 6748 | 2 | 20.47 | 6 | .002 |
| **Age 12** |  |  |  |  |  |  |  |
| 0 Saturated | 52 | 10447.34 | 4913 | - | - | - | - |
| 1 Saturated: ST = DT | 37 | 10509.68 | 4928 | 0 | 62.34 | 15 | <.001 |
| *2 ACE* | *23* | *10461.64* | *4942* | *0* | *14.30* | *29* | *.990* |
| 3 ACE: Boys = Girls | 15 | 10538.20 | 4950 | 2 | 76.56 | 8 | <.001 |
| 4 ACE: ST = DT | 17 | 10509.94 | 4948 | 2 | 48.30 | 6 | <.001 |
| 5 ACE: Correlated Errors | 18 | 10515.73 | 4947 | 0 | 59.45 | 33 | .003 |
| 6 ACE: FT = MT | 17 | 10498.14 | 4948 | 2 | 36.50 | 6 | <.001 |

^FT = female teacher; MT = male teacher; DT = different teacher; ST = same teacher; ep = estimated parameters; df =^

^degrees of freedom; -2ll = -2loglikelihood; A = additive genetic effects; C = common environmental effects; E = unique^

^environmental effects^

**Table S3** Genetic modeling results for the hyperactivity (HYP) scale

|  | **ep** | **-2ll** | **df** | **model** | **χ2** | **Δdf** | **p** |
| --- | --- | --- | --- | --- | --- | --- | --- |
| **Age 7** |  |  |  |  |  |  |  |
| 0 Saturated | 52 | 20030.50 | 7374 | - | - | - | - |
| 1 Saturated: ST = DT | 37 | 20187.51 | 7389 | 0 | 157.01 | 15 | <.001 |
| 2 ADE | 23 | 20063.58 | 7403 | 0 | 33.08 | 29 | .275 |
| 3 ADE: Boys = Girls | 15 | 20102.04 | 7411 | 2 | 38.46 | 8 | <.001 |
| 4 ADE: ST = DT | 17 | 20199.68 | 7409 | 2 | 136.10 | 6 | <.001 |
| 5 ADE: Correlated Errors | 18 | 20085.22 | 7408 | 0 | 54.00 | 33 | .012 |
| *6 ADE: FT = MT* | *17* | *20078.37* | *4709* | *2* | *14.79* | *6* | *.022* |
| **Age 9** |  |  |  |  |  |  |  |
| 0 Saturated | 52 | 17649.84 | 6709 | - | - | - | - |
| 1 Saturated: ST = DT | 37 | 17783.59 | 6724 | 0 | 133.76 | 15 | <.001 |
| 2 ADE | 23 | 17681.08 | 6738 | 0 | 31.24 | 29 | .354 |
| 3 ADE: Boys = Girls | 15 | 17707.93 | 6746 | 2 | 26.84 | 8 | .001 |
| 4 ADE: ST = DT | 17 | 17793.64 | 6744 | 2 | 112.56 | 6 | <.001 |
| 5 ADE: Correlated Errors | 18 | 17704.15 | 6743 | 0 | 53.63 | 33 | .013 |
| *6 ADE: FT = MT* | *17* | *17697.01* | *6744* | *2* | *15.92* | *6* | *.014* |
| **Age 12** |  |  |  |  |  |  |  |
| 0 Saturated | 52 | 12142.50 | 4917 | - | - | - | - |
| 1 Saturated: ST = DT | 37 | 12258.51 | 4932 | 0 | 117.01 | 15 | <.001 |
| *2 ADE* | *23* | *12176.31* | *4946* | *0* | *33.81* | *29* | *.246* |
| 3 ADE: Boys = Girls | 15 | 12219.40 | 4954 | 2 | 43.10 | 8 | <.001 |
| 4 ADE: ST = DT | 17 | 12249.56 | 4952 | 2 | 73.26 | 6 | <.001 |
| 5 ADE: Correlated Errors | 18 | 12216.43 | 4951 | 0 | 74.35 | 33 | <.001 |
| 6 ADE: FT = MT | 17 | 12204.84 | 4952 | 2 | 28.53 | 6 | <.001 |

^FT = female teacher; MT = male teacher; DT = different teacher; ST = same teacher; ep = estimated parameters; df = degrees of^

^freedom; -2ll = -2loglikelihood; A = additive genetic effects; D = additive genetic effects; E = unique environmental effects^

**Table S4** Genetic modeling results for the ADHD index (ADHD) scale

|  | **ep** | **-2ll** | **df** | **model** | **χ2** | **Δdf** | **p** |
| --- | --- | --- | --- | --- | --- | --- | --- |
| **Age 7** |  |  |  |  |  |  |  |
| 0 Saturated | 52 | 24482.63 | 7369 | - | - | - | - |
| 1 Saturated: ST = DT | 37 | 24614.63 | 7384 | 0 | 132.00 | 15 | <.001 |
| 2 ADE | 23 | 24513.40 | 7398 | 0 | 30.77 | 29 | .376 |
| *3 ADE: Boys = Girls* | *15* | *24533.14* | *7406* | *2* | *19.73* | *8* | *.011* |
| 4 ADE: ST = DT | 12 | 24640.09 | 7409 | 3 | 106.95 | 3 | <.001 |
| 5 ADE: Correlated Errors | 13 | 24549.49 | 7408 | 3 | 59.55 | 38 | .014 |
| 6 ADE: FT = MT | 12 | 24546.27 | 7409 | 3 | 13.13 | 3 | .004 |
| **Age 9** |  |  |  |  |  |  |  |
| 0 Saturated | 52 | 22137.31 | 6703 | - | - | - | - |
| 1 Saturated: ST = DT | 37 | 22271.04 | 6718 | 0 | 133.72 | 15 | <.001 |
| 2 ADE | 23 | 22159.55 | 6732 | 0 | 22.24 | 29 | .810 |
| 3 ADE: Boys = Girls | 15 | 22174.92 | 6740 | 2 | 15.37 | 8 | .052 |
| 4 ADE: ST = DT | 12 | 22274.78 | 6743 | 3 | 99.85 | 3 | <.001 |
| 5 ADE: Correlated Errors | 13 | 22197.56 | 6742 | 0 | 60.25 | 38 | .012 |
| *6 ADE: FT = MT* | *12* | *22176.08* | *6743* | *3* | *1.15* | *3* | *.765* |
| **Age 12** |  |  |  |  |  |  |  |
| 0 Saturated | 52 | 15589.30 | 4912 | - | - | - | - |
| 1 Saturated: ST = DT | 37 | 15704.31 | 4927 | 0 | 115.02 | 15 | <.001 |
| 2 ADE | 23 | 15624.83 | 4941 | 0 | 35.53 | 29 | .188 |
| 3 ADE: Boys = Girls | 15 | 15638.42 | 4949 | 2 | 13.59 | 8 | .093 |
| 4 ADE: ST = DT | 12 | 15733.73 | 4952 | 3 | 95.30 | 3 | <.001 |
| 5 ADE: Correlated Errors | 13 | 15679.13 | 4951 | 0 | 89.60 | 38 | <.001 |
| *6 ADE: FT = MT* | *12* | *15645.36* | *4952* | *3* | *6.94* | *3* | *.074* |

^FT = female teacher; MT = male teacher; DT = different teacher; ST = same teacher; ep = estimated parameters; df = degrees of^

^freedom; -2ll = -2loglikelihood; A = additive genetic effects; D = additive genetic effects; E = unique environmental effects^
